# Antiviral Action against SARS-CoV-2 of a Synthetic Peptide Based on a Novel Defensin Present in the Transcriptome of the Fire Salamander (*Salamandra salamandra*)

**DOI:** 10.3390/pharmaceutics16020190

**Published:** 2024-01-29

**Authors:** Ana Luisa A. N. Barros, Vladimir C. Silva, Atvaldo F. Ribeiro-Junior, Miguel G. Cardoso, Samuel R. Costa, Carolina B. Moraes, Cecília G. Barbosa, Alex P. Coleone, Rafael P. Simões, Wanessa F. Cabral, Raul M. Falcão, Andreanne G. Vasconcelos, Jefferson A. Rocha, Daniel D. R. Arcanjo, Augusto Batagin-Neto, Tatiana Karla S. Borges, João Gonçalves, Guilherme D. Brand, Lucio H. G. Freitas-Junior, Peter Eaton, Mariela Marani, Massuo J. Kato, Alexandra Plácido, José Roberto S. A. Leite

**Affiliations:** 1 Núcleo de Pesquisa em Morfologia e Imunologia Aplicada, NuPMIA, Faculdade de Medicina, Universidade de Brasília, UnB, Brasília 70910-900, DF, Brazil; analuisaanbarros@gmail.com (A.L.A.N.B.); atvaldo.junior@aluno.unb.br (A.F.R.-J.); mgcardoso@ff.ulisboa.pt (M.G.C.); wanessa.felix@unb.br (W.F.C.); andreannegv@gmail.com (A.G.V.); tatianakarlab@gmail.com (T.K.S.B.); 2 Programa de Pós-graduação em Medicina Tropical, PGMT, Faculdade de Medicina, Universidade de Brasília, UnB, Brasília 70910-900, DF, Brazil; 3 Laboratório de Vigilância Genômica e Biologia Molecular-Fundação Oswaldo Cruz Piauí, Teresina 64001-350, PI, Brazil; vladimir.costa@fiocruz.br; 4 imed.ULisboa-Research Institute for Medicines, Faculty of Pharmacy, University of Lisbon, 1649-003 Lisbon, Portugal; jgoncalv@ff.ulisboa.pt; 5 Instituto de Química, IQ, Universidade de Brasília, UnB, Brasília 70910-900, DF, Brazil; samuel.r.costa@hotmail.com (S.R.C.); gdbrand@gmail.com (G.D.B.); 6 Department of Pharmaceutical Sciences, Federal University of São Paulo, Diadema 09913-030, SP, Brazil; carolinaborsoi@gmail.com; 7 Department of Microbiology, Institute of Biomedical Sciences, University of Sao Paulo, São Paulo 05508-000, SP, Brazil; cecigomes.barbosa@gmail.com (C.G.B.); luciofreitasjunior@gmail.com (L.H.G.F.-J.); 8 Programa de Pós-Graduação em Ciência e Tecnologia de Materiais (POSMAT), School of Sciences, São Paulo State University (UNESP), Bauru 17033-360, SP, Brazil; alex.coleone@unesp.br (A.P.C.); netobat@gmail.com (A.B.-N.); 9 School of Agriculture, Department of Bioprocess and Biotechnology, São Paulo State University (UNESP), Botucatu 18618-689, SP, Brazil; rafael.simoes@unesp.br; 10 Bioinformatics Postgraduate Program, Metrópole Digital Institute, Federal University of Rio Grande do Norte, Natal 59078-900, RN, Brazil; raul.maia.089@ufrn.edu.br; 11 People&Science Pesquisa Desenvolvimento e Inovação LTDA, Centro de Desenvolvimento Tecnológico (CDT), Universidade de Brasília, UnB, Brasília 70910-900, DF, Brazil; 12 Campus São Bernardo, Universidade Federal do Maranhão, UFMA, São Bernardo 65550-000, MA, Brazil; jeffersonbiotec@gmail.com; 13 Department of Biophysics and Physiology, Federal University of Piauí, Teresina 64049-550, PI, Brazil; daniel.arcanjo@ufpi.edu.br; 14 Institute of Sciences and Engineering, São Paulo State University (UNESP), Itapeva 18409-010, SP, Brazil; 15 Laboratório Associado para a Química Verde/Rede de Química e Tecnologia (LAQV/REQUIMTE), Departamento de Química e Bioquímica, Faculdade de Ciências, Universidade do Porto, 4169-007 Porto, Portugal; pete.eaton@gmail.com (P.E.); alexandra.nascimento@fc.up.pt (A.P.); 16 School of Chemistry, The Bridge, University of Lincoln, Lincoln LN6 7EL, UK; 17 IPEEC-CONICET, Consejo Nacional de Investigaciones Científicas y Técnicas, Puerto Madryn 9120, Argentina; mmarani@cenpat-conicet.gob.ar; 18 Instituto de Química (IQ), Universidade de São Paulo (USP), São Paulo 05508-900, SP, Brazil; massuojorge@gmail.com

**Keywords:** amphibians, transcriptomics, antimicrobial peptides, bioinformatics, antiviral action, SARS-CoV-2 infection

## Abstract

The potential emergence of zoonotic diseases has raised significant concerns, particularly in light of the recent pandemic, emphasizing the urgent need for scientific preparedness. The bioprospection and characterization of new molecules are strategically relevant to the research and development of innovative drugs for viral and bacterial treatment and disease management. Amphibian species possess a diverse array of compounds, including antimicrobial peptides. This study identified the first bioactive peptide from *Salamandra salamandra* in a transcriptome analysis. The synthetic peptide sequence, which belongs to the defensin family, was characterized through MALDI TOF/TOF mass spectrometry. Molecular docking assays hypothesized the interaction between the identified peptide and the active binding site of the spike WT RBD/hACE2 complex. Although additional studies are required, the preliminary evaluation of the antiviral potential of synthetic SS-I was conducted through an in vitro cell-based SARS-CoV-2 infection assay. Additionally, the cytotoxic and hemolytic effects of the synthesized peptide were assessed. These preliminary findings highlighted the potential of SS-I as a chemical scaffold for drug development against COVID-19, hindering viral infection. The peptide demonstrated hemolytic activity while not exhibiting cytotoxicity at the antiviral concentration.

## 1. Introduction

The indiscriminate utilization of antimicrobial agents and, as a consequence, antimicrobial-resistant pathogens pose a significant threat to the effectiveness of treating prevalent infectious diseases [1]. In addition, the recent rise of zoonotic diseases, including severe acute respiratory syndrome coronavirus (SARS-CoV) and Middle East respiratory syndrome coronavirus (MERS-CoV) [2], has exacerbated the situation due to the absence of appropriate resources for the rapid and effective development of new drugs.

Resistance to conventional antibiotics is a major global public health concern, leading to prolonged hospitalization times and a high mortality rate with consequent economic impacts.

Similar to other coronaviruses, SARS-CoV-2 relies on the surface spike glycoprotein to access the host cells, mainly through the interaction between its receptor-binding domain (RBD) and the host cell receptor angiotensin-converting enzyme 2 (ACE2) [3,4,5]. SARS-CoV-2 infection triggers a deep downstream pro-inflammatory cytokine storm. Elevated levels of pro-inflammatory cytokines can result in detrimental tissue damage, including lung tissue damage, respiratory failure, and, ultimately, multi-organ failure in COVID-19 patients. Therefore, molecular entities that can interfere with the binding of the SARS-CoV-2 spike protein to ACE2 have the potential to inhibit viral entry, thus reducing viral infectivity.

One promising approach to blocking viral activity is the application of natural peptide molecules due to their general advantages, such as high specificity and effectiveness. They are also characterized by easy and rapid production (despite high costs), low toxicity (minimal side effects and easy metabolism), and a particular mechanism of action that delays the emergence of resistant mutants. Moreover, natural peptides can be used synergistically with other drugs [6]. Natural peptides can serve as models for the development of modified synthetic antimicrobial peptides (AMPs). Peptides are highly versatile and amenable to improvement through design, which allows limitations to be addressed, such as a short half-life or poor oral absorption [7,8]. As a result, numerous peptide-based drugs are currently commercially available for treating numerous ailments, such as hepatitis C, myeloma, skin infections, and diabetes [9], with several hundred more in either the preclinical or clinical stage of development [10,11].

AMPs have been isolated from most life forms and are categorized into different peptide families according to their particular sequences and structures. The defensin AMP family is identified as abundant cysteine-rich AMPs divided into three groups, including α-, β-, and θ-defensins, based on the differential connections of their three disulfide bridges. Defensins and defensin-like peptides have been identified in fungi [12], plants [13], and numerous animals [14], including humans [15]. These peptides have demonstrated antimicrobial activity against multiple microorganisms [14,16,17,18,19].

Amphibians are an important source of AMPs that possess the ability to neutralize or kill microorganisms [20]. They present granular glands in their skin that synthesize and secrete a diverse array of peptides as part of their innate immune defense system. These AMPs, which vary in structure and size, exhibit a diversity of bioactivities, including antiviral, antifungal, anticancer, antioxidant, immune modulation, and inflammation responses. Moreover, they employ various mechanisms of action [21]. Each anuran species thus far exhibits a specific composition of peptides, although many of these peptides display similarities among closely related species, highlighting their common origin [22]. The widespread European fire salamander, *Salamandra salamandra* [23], is characterized by its conspicuously yellow and black skin and possesses specific glands that produce a defensive poison. The secretion of salamanders has been found to contain a variety of compounds [24], including peptides and alkaloids [25] with toxic, antimicrobial [14,26], and antioxidant properties [27]. The bioprospection and characterization of new molecules from amphibian species are of utmost strategic relevance to provide essential tools for immediate use in the research and development of drugs for viral and bacterial treatment and management. It is crucial to encourage academic endeavors in this direction, considering that, between 2015 and 2019, several of the new peptide-based drugs accepted by the FDA came about from the efforts of academic groups [10].

Here, we report the description and characterization of a new β-defensin-bioactive peptide identified from the transcriptome analysis of *S. salamandra.* We describe the 3D molecular docking in silico assay of its interaction with the complex spike WT RBD/hACE2. Moreover, we assess the SS-I synthetic peptide antiviral action through a Vero-CCL-81 cell-based SARS-CoV-2 infection test and its cytotoxic and hemolytic effects.

## 2. Materials and Methods

### 2.1. Transcriptome Assembly and Identification of Transcript Sequence by Homology

An RNA-seq dataset of *S. salamandra* was obtained from the Sequence Read Archive (SRA) under accession number SRR11118085. This database was derived from the collection, processing of biological material, and gene identification obtained from the tail-tip of the *S. salamandra* salamander [28]. The transcriptome data in this dataset were generated using paired-end total RNA sequencing (Illumina HiSeq2000, San Diego, CA, USA), and microarrays utilized for gene expression analysis were designed by Czypionka et al. [29].

Raw-read quality control and adapter trimming were carried out, respectively, in FastQC (v0.11.4) [30] and Trim Galore (v0.4.1) [31] using the option to execute Cutadapt (v1.8.3) [32] with default values. The reads were assembled using rnaSPAdes (v3.14.0) [33] without the read error correction mode (only the assembler parameter). Finally, a database was created from the assembled transcripts using makeblastdb (v2.6.0) [34], followed by a local tblastn search using the CFBD-1 defensin sequence from *C. fudingensis* as the query [14]. The best hit transcript obtained from the search was selected for further analysis. The Expasy translation tool was used to confirm the findings (https://web.expasy.org/translate/ (accessed on 23 April 2023)). Multiple sequence alignment was performed between the identified ORFs and amino acid sequences with the *C. fundingensis* sequence to assess identity and homology.

### 2.2. Sequence Alignment and Phylogenetic Analysis

A protein-BLAST (BLASTp) search [35] was conducted using the CFBD-1 amino acid sequence as the query against the non-redundant protein sequence database at the NCBI (National Center for Biotechnology Information). The search utilized default parameters. From the BLAST results, the 12 sequences with the lowest E values were selected for further analysis. The selected sequences were then subjected to the multiple sequence alignment program Clustal Omega [36] from the European Molecular Biology Laboratory—European Bioinformatics Institute (EMBL-EBI), employing default settings. For the phylogenetic tree, the results were visualized using EvolView [37,38,39]. To facilitate alignment visualization, Jalview 2.11.2.6 [40] was employed. In the alignment, the conserved amino acids, meaning those with more than 50% identity at a specific position, were highlighted using the Clustal coloring method. The sequences were organized into four groups, each sorted by similarity to SS-I. The first group comprises the sequences of SS-I and CFBD-1. The second group includes all the β-defensin peptides. The third group consists of the identified hypothetical proteins, while the fourth group comprises β-defensin-unrelated peptides.

### 2.3. In Silico Studies

#### 2.3.1. Docking

The structural model of SS-I was generated using homology modeling with the assistance of the MPI Bioinformatics Toolkit [41,42], and subsequent refinement was performed using the PyMOL software, version 2.5 [43]. The protonation states of the residues were evaluated at neutral pH using the PDB2PQR server [44].

The protein–peptide interactions between hACE2 and SS-I (ACE2-PEP), as well as the binding structure of SS-I and SARS-CoV-2 spike RBD (SPIKE-PEP), were evaluated through protein–peptide flexible docking using the HADDOCK2.4 server [45,46]. The ACE2 and spike structures were obtained from Protein Data Bank files (6LZG) [47] and isolated via the PyMOL software. Active interfacial residues at the ACE2/SPIKE were selected for docking analysis.

#### 2.3.2. Molecular Dynamics Simulations

Molecular dynamics (MD) simulations were performed using the conformational structure complexes obtained in the previous step of molecular docking, henceforth referred to as ACE2-PEP-S1 and ACE2-PEP-S2 (representing the two best solutions from the docking between the ACE2 protein and the peptide of interest) and SPIKE-PEP-S1 and SPIKE-PEP-S2 (representing the two best solutions from the docking between the spike protein and the peptide of interest). All MD simulations were conducted using the GROMACS v.2018 software [48]. The protonation states of the amino acid residues at pH 7.0 were evaluated using the PDB2PQR server [44]. The structures of the ACE2-PEP-S1, ACE2-PEP-S2, SPIKE-PEP-S1, and SPIKE-PEP-S2 complexes were energetically optimized using a combination of steepest descent (SD) and adopted basis Newton–Raphson (ABNR)-based energy minimization (500 steps). Subsequently, the complexes were subjected to a 100 ps MD simulation for temperature equilibration at 300 K, using the Charmm force field [49] with explicit solvent (TIP3P). Then, 100 ns MD simulations were performed under a constant temperature (T = 300 K) and pressure (1.0 bar), also employing the Charmm force field. The stability of the complexes during the MD simulations was evaluated by analyzing parameters between the molecules’ interfacing residues, such as residues making hydrogen/disulfide bonds, salt bridges, and covalent links. These analyses were performed using the tools of the PDBePISA web service [50] and the VMD software, version 1.9.3 [51]. Hydrogen bonds were considered present if the donor–acceptor distance was approximately 3.5 Å, the angle was around 30°, and the salt-bridge cutoff was <4.0 Å [52]. Two-dimensional ligand–protein interaction diagrams were made using the LigPlot+ web service [53].

#### 2.3.3. Electronic Structure Calculations

The initial (pre-optimized) structures employed in the MD of ACE2-PEP-S1, ACE2-PEP-S2, SPIKE-PEP-S1, and SPIKE-PEP-S2 were employed as inputs for the calculation of local chemical reactivity descriptors. Four structures with slightly different conformations were evaluated using the density functional theory (DFT) framework. The B3LYP exchange–correlation functional [54,55,56] and the 6-31G(d,p) basis set were applied to all atoms. The calculations were performed in water, utilizing the polarizable continuum model (PCM) [57].

Chemical reactivity was assessed using condensed-to-atoms Fukui indexes (CAFIs) [58]. These indexes provide valuable information about the reactivity of peptide sites towards nucleophilic (*f*^+^) or electrophilic (*f*^−^) agents and have been widely used in peptide studies [27,59,60]. The CAFIs were calculated based on finite differences in the atomic populations obtained through Hirshfeld’s partition method, following a procedure described elsewhere [61,62].

### 2.4. Synthesis and Characterization of Peptide

The peptide SS-I was synthesized manually, with a standard Fmoc (N-(9-fluorenyl)methoxycarbonyl) chemistry. The synthesis was initiated with 215 mg of resin (0.70 mmol/g, Peptides International). Fmoc-protected amino acids (Peptides International) were used in a four-fold to six-fold molar excess relative to the nominal synthesis scale (1.2 mmol). Couplings were performed with 1,3-diisopropyl carbodiimide/ethyl 2-cyano-2-(hydroxyimino) acetate (DIC/Oxyma^®️^) in N,N-dimethylformamide (DMF) for 2–3 h. Amino group deprotections were carried out using a mixture of 4-methyl piperidine/DMF (1:4, *v*:*v*) for 20–30 min. Each deprotection and coupling were confirmed through a Kaiser test. The cleavage of the peptide from the resin and the removal of side-chain protecting groups were performed using 10.0 mL of a cleavage cocktail (Reagent K), shaken at room temperature for 90 min [63,64]. After solvent evaporation under nitrogen, the peptide was precipitated by adding cold diisopropyl ether, collected through filtration, and washed four times with the same solvent. Extraction was performed using a mixture of 200 mL of water and acetonitrile (1:1, *v*:*v*), and the crude peptide was lyophilized. The synthesized products were purified using reverse-phase HPLC with a C18 column (250 × 20 mm i.d., 15 μm, Shim-Pack PREP-ODS). To confirm the presence of the purified molecules, MALDI-TOF/TOF mass spectrometry (Ultraflex III Extreme Bruker Daltonics) was used in the positive, reflector mode [65].

### 2.5. In Vitro Synthetic SS-I Viability Evaluation

C20 cells were cultured in a DMEM-F12 medium supplemented with L-glutamine, 10% FBS, and 1% Pen/Strep at 37 °C and 5% CO_2_. For the experiments, 5 × 10^4^ cells were seeded in each well. After four hours, the cells were treated with different concentrations of the SS-I synthetic peptide (5, 10, 50, and 100 μM). The cells were then incubated for 24 h.

For the MTT assay, 100 μL of 3-(4,5-dimethylthiazol-2-yl)-2,5-diphenyltetrazolium bromide (MTT) at 0.5 mg/mL was added to the cells. After four hours of incubation with MTT, DMSO was added to the wells to solubilize the formazan crystals, and the plate was measured at 570 nm. Cells treated with 30% DMSO were used as the positive control. The viability percentage was calculated through normalization to the basal value. Statistical analyses of five independent experiments were performed using GraphPad Prism version 9.5 (GraphPad Software, San Diego, CA, USA), including Brown–Forsythe and Welch ANOVA tests associated with Dunnett’s T3 multiple comparisons test.

### 2.6. In Vitro Vero-CCL-81 Cell-Based SARS-CoV-2 Infection Assay

The purified SS-I synthetic peptide was initially diluted to a concentration of 2 mM in DMSO and subsequently diluted in PBS to achieve a concentration of 60 μM. From this prepared working solution, 10 μL was added to the assay plates. The initial concentration for the dose–response assay was set at 10 μM. DMSO-treated infected cells and DMSO-treated non-infected cells were used as positive and negative controls, respectively.

Vero CCL-81 cells were plated in a 384-well plate and, after 24 h, the compounds were added to the cells previously described. Following that, the SARS-CoV-2 virus (SP02/human/2020/BR; GenBank Accession No. MT126808.1) was added at a multiplicity of infection of 0.1 viral particles per cell. The final DMSO concentration in the assay was 0.5% (*v*/*v*). After 33 h, the cells were fixed in 4% paraformaldehyde (in PBS pH 7.4) and immunofluorescence was performed using serum from a convalescent COVID-19 patient diluted at 1:1000 in 5% bovine serum albumin (BSA) in PBS, which served as the primary antibody. After 30 min, the wells were washed. A solution of Alexa488-conjugated goat anti-human IgG (Thermo Fisher Scientific, Waltham, MA, USA), and 5 µg/mL of DAPI (4′,6 diamidino-2-phenylindole; Sigma-Aldrich, St. Louis, MO, USA) diluted at 1:1000 in 5% BSA (*v*/*v*) was added to each well and incubated for 30 min. All wells were washed twice with PBS, and images were acquired using the HCS Operetta (PerkinElmer, Waltham, MA, EUA) and subsequently analyzed with the Harmony software (Perkin Elmer, Waltham, MA, USA), version 3.5.2. The measured parameters in each well were the total cell number and the total number of infected cells. The ratio between the number of infected cells and the total cell number was defined as the infection rate (IR). The antiviral activity was calculated based on the IR normalization to the negative control (DMSO-treated infected cells). The cell survival ratio was calculated by normalizing the total cell number in each well to the average number of cells in the positive control wells. A nonlinear regression analysis and sigmoidal dose–response (variable slope) were performed using GraphPad Prism version 7.0, considering three independent experiments.

### 2.7. Hemolysis Assay

To assess the hemolytic activity of synthetic SS-I, 4 mL of whole blood was collected in EDTA (1.8 mg/mL) tubes and then centrifuged at 1500 rpm for 10 min. Red blood cells (RBCs) were washed three times with PBS (pH 7.2) at 37 °C, replacing the removed plasma volume. A two-fold serial dilution of synthetic SS-I was prepared in a round-bottom plate starting at 100 μM. Then, 75 μL of 10% RBCs in PBS was added to each well containing 75 μL of the sample, PBS (negative control), or 0.1% Triton-X (positive control), and mixed carefully. All concentrations and controls were tested in triplicate. The plate was incubated for 1 h at 37 °C under gentle agitation. After the incubation, the plate was centrifuged at 4500 rpm for 5 min, 100 μL was transferred to a new flat-bottom plate, and finally measured at 550 nm. *Hemolytic activity* (%) = (Abs _sample_ − Abs _PBS_)/(Abs _Triton-X_ − Abs _PBS_) × 100.

## 3. Results and Discussion

### 3.1. Identification and Characterization of the Defensin SS-I

**Figure 1:**
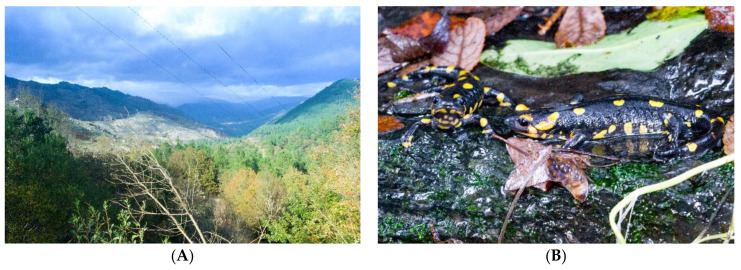
(**A**) Peneda-Gerês National Park, Portugal. (**B**) Specimens of fire salamanders (*S. salamandra*). Photo credit: Peter Eaton.

The identification of CFBD-1 as the first defensin with antimicrobial properties from *Cynops fudingensis* [14] and the subsequent discovery of Salamandrin-I from *S. salamandra* [27], where a sequence similarity between these two peptides was described by Plácido et al., was the starting point for this work. *C. fudingensis*, commonly known as the Fuding fire-bellied newt, has been described to live predominantly in an aquatic environment. Contrarily, fire salamanders are known to live in hilly forest areas (Figure 1). When compared to its aquatic counterpart, the terrestrial environment is associated with higher UV radiation levels and more significant variations in temperature and humidity. Besides the selection pressure on the animal species to adapt better to their settings, microbial pathogens undergo thorough adaptative evolution [66]. The conditions where fire salamanders live have been proven to provide these amphibians with antioxidant peptides (AOPs) and AMPs with much richer diversity and greater potency [67]. Considering this, the strategy was to use the CFBD-1 sequence to identify a similar peptide in the *S. salamandra* RNA-seq dataset through bioinformatic prospection.

**Figure 2:**
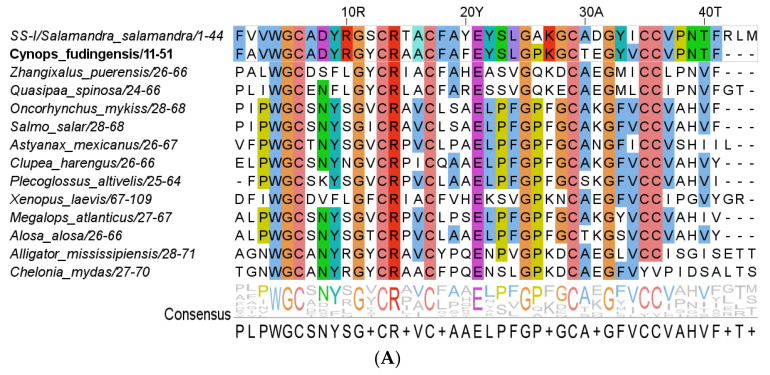

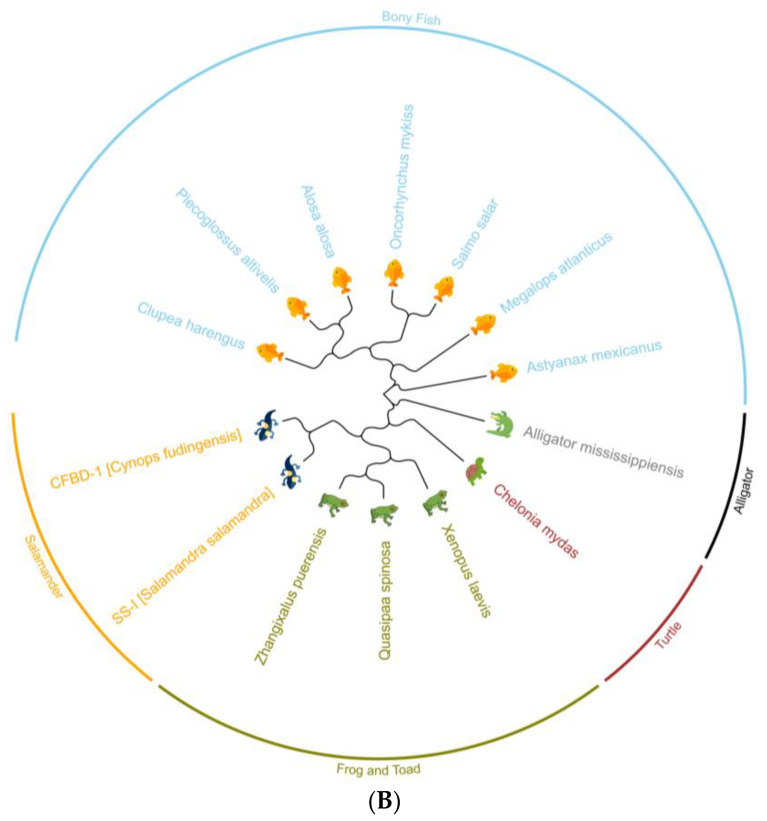
Results of BLASTp analysis. (**A**) Multiple alignment was performed using the Clustal Omega tool. The sequences were categorized into four groups, each sorted by similarity to SS-I. The CFDB-1 peptide, highlighted in bold, was used to identify SS-I. The first group comprises the sequences of SS-I and CFBD-1 (gray-lined box). In this group, conserved amino acids are colored. The second group consists of β-defensin peptides spanning from *Zhangixalus puerensis* to *Plecoglossus altivelis*. The third group includes hypothetical proteins of *Xenopus laevis*, *Megalops atlanticus*, and *Alosa alosa*. The fourth group encompasses species whose peptides do not belong to the β-defensin family. For all sequences, the conserved amino acids, meaning those present in more than 50% of the sequences at a specific position, were colored. The numbers following the species name indicate the aligned sequence limits. (**B**) Phylogenetic tree generated from the Clustal Omega alignment results.

A novel β-defensin antimicrobial peptide, named SS-I, was identified from the tail-tip of *S. salamandra* larvae by combining RNA sequencing and bioinformatics. A BLAST search revealed a significant sequence similarity between SS-I and several β-defensin AMPs from other animals (Figure 2A). The multiple alignment was sorted by similarity to SS-I, with the aligned sequences from *Zhangixalus puerensis* to *Plecoglossus altivelis* representing β-defensin peptides. The displayed sequences from *Xenopus laevis*, *Megalops atlanticus*, and *Alosa alosa* correspond to hypothetical proteins. During genome sequencing, it is common to identify open reading frames (ORFs) that encode proteins that have not yet been proven to be expressed in the organism or whose function remains unknown.

Since these identified hypothetical proteins exhibit high similarity to other β-defensin peptides, it is plausible that they may share a similar function. However, it should be noted that similarity and the presence of multiple conserved residues do not directly imply a functional association. For instance, the peptide sequences of *Alligator mississipiensis* and *Chelonia mydas* represent an ADAM9-like (disintegrin and metalloproteinase domain-containing protein 9-like) protein and POLD3 (DNA polymerase delta subunit 3), respectively. Despite exhibiting highly conserved amino acids, such as glycine and cysteine, which play an important role in determining peptide structure, their functional connection remains unclear. Figure 2B shows a clear separation between exclusively aquatic species and those capable of exploiting both aquatic and terrestrial habitats. Moreover, it is apparent that *S. salamandra* exhibits a closer phylogenetic relationship with frogs and toads, followed by turtles and, lastly, alligators.

**Figure 3:**
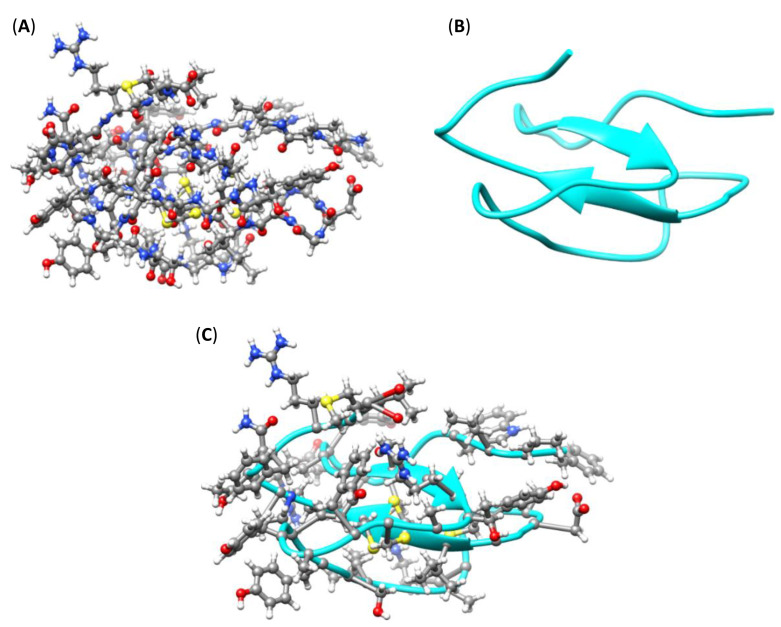
Hypothesized representations of SS-I peptide. (**A**) Ball-and-stick representation. (**B**) Ribbon representation. (**C**) Overlap of ball-and-stick and ribbon diagrams.

Figure 3 illustrates the homology-predicted 3D structure of the identified mature peptide consisting of 44 residues. The complete sequence of the peptide is NH_2_-FVVWGCADYRGSCRTACFAYEYSLGAKGCADGYICCVPNTFRLM-COOH, which contains six cysteines, four basic, and three acidic residues. The theoretical mass of the peptide is [M + H]^+^ (average mass) 4870.684 and [M + H]^+^ (monoisotopic) 4867.1515 [68]. The predicted peptide identified in the transcriptome analysis was synthesized, purified, and characterized via mass spectrometry for subsequent use in biological assays, as per the section on the materials and methods. The predicted structure presents disulfide bonds that define its global conformation. However, after the peptide synthesis, the cysteine oxidation was unsuccessful; thus, the correct folding was not attained, as the disulfide bonds were not formed.

### 3.2. Interactions between SS-I with ACE2 and SARS-CoV-2 Spike Protein (S1)

The spike protein of SARS-CoV-2 comprises two subunits, S1 and S2, which play a two-step process: (i) receptor recognition and (ii) cell membrane fusion. The S1 subunit contains a receptor-binding domain that recognizes and binds to the host receptor, ACE2, while the S2 subunit mediates viral cell membrane fusion by forming a six-helical bundle via the two-heptad repeat domain [69,70]. S1 can be divided into an N-terminal domain (NTD) and a C-terminal domain (CTD), and SARS-CoV-2 utilizes the S1 CTD to recognize the receptor (also called the receptor-binding domain (RBD)) [71]. In this way, the RBD region is a critical target for neutralizing SARS-CoV-2. For all explained here, we use a 2.5 Å crystal structure of SARS-CoV-2-CTD in complex with ACE2 (S1 subunit), reported in a previous study [71], to perform our analysis. In this study, we are also interested in identifying possible interactions between the proposed peptide (PEP) and the ACE2 cell receptor. Thus, as explained in more detail in the methodology, the docking solutions were identified as two different complexes: (i) the peptide complexed with the cell receptor (ACE2-PEP) and (ii) the peptide complexed with the SARS-CoV-2-CTD (SPIKE-PEP). The molecular complexes obtained through the docking and used for MD simulations (ACE2-PEP-S1, ACE2-PEP-S2, SPIKE-PEP-S1, and SPIKE-PEP-S2) are presented in the supplementary material (Figure S1). Here, S1 and S2 represent the two best docking solutions, respectively, for each complex.

**Figure 4:**
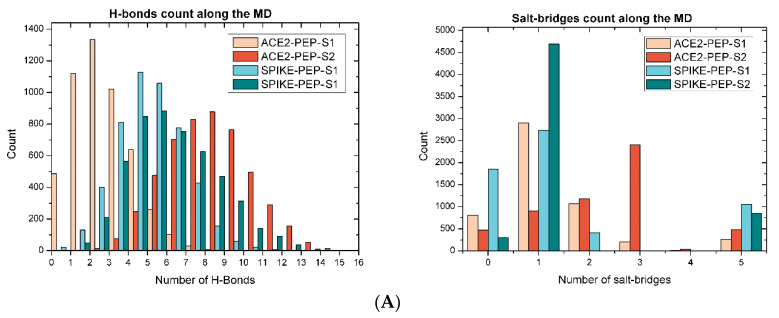

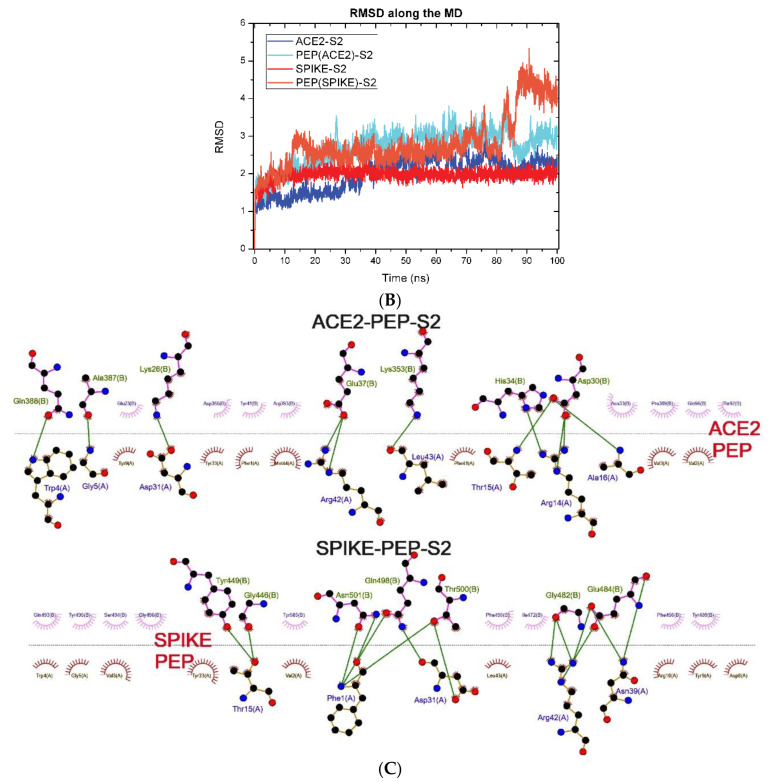
(**A**) Frequency of H-bonds and salt bridges during MD simulations in the ACE2-PEP-S1, ACE2-PEP-S2, SPIKE-PEP-S1, and SPIKE-PEP-S2 complexes. (**B**) Root-mean-square deviation (RMSD) from MD simulations of the two best docking solutions: ACE2-PEP-S2 and SPIKE-PEP-S2. RMSD values are presented for each protein: ACE2, PEP(ACE2) (peptide complexed with ACE2 protein), SPIKE, and PEP(SPIKE) (peptide complexed with spike. (**C**) Two-dimensional ligand–protein interaction diagrams for the complexes ACE2-PEP-S2 and SPIKE-PEP-S2.

Protein–protein interactions are essentially stabilized by hydrogen bonds (H-bonds), disulfide bonds, salt bridges, and, in rare cases, covalent links [72]. In the case of the molecular complexes under study, only hydrogen bonds and salt bridges were identified between the interface residues. Therefore, the analysis presented in Figure 4A shows significant differences in the number of H-bonds observed during the MD simulations of the complexes. The interactions between the proteins on the complexes ACE2-PEP-S1 and SPIKE-PEP-S1 presented a low frequency of H-bonds between zero and three, which makes the interaction between the proteins of the complex quite unstable. Conversely, the ACE2-PEP-S2 and SPIKE-PEP-S2 complexes showed a higher frequency of H-bonds, predominantly ranging between four and ten, despite the peptide consisting of only 44 amino acids. Similar behavior was observed for the frequency of salt bridges monitored throughout the simulation. Figure 4A shows that the ACE2-PEP-S2 complex has a predominant frequency of salt bridges equal to three, while the ACE2-PEP-S1 complex has a predominant frequency equal to one. In the case of the complexes containing the SPIKE protein, it was observed that both the SPIKE-PEP-S1 and SPIKE-PEP-S2 complexes have a predominant frequency of one. However, the SPIKE-PEP-S1 complex also showed a significant frequency of 0; that is, this complex remains for a considerable time during simulation without making salt bridge interactions. Although these docking solutions are energetically viable, these results suggest that the formation of the complexes ACE2-PEP-S1 and SPIKE-PEP-S1 may not be promising. On the other hand, these results suggest that the ACE2-PEP-S2 and SPIKE-PEP-S2 complexes are more stable, evidencing that the peptide can interact effectively with both the spike and the ACE2 proteins.

H-bonds and salt bridges with an occupancy higher than 10% during the MD between residues of the ACE2-PEP-S2 and SPIKE-PEP-S2 complexes are presented in Table 1. These residues play a critical role in maintaining the stability of the complexes. Figure 4B shows the root-mean-square deviation (RMSD) of atomic positions obtained from the MD simulations. The RMSD values are presented independently for each protein: ACE2, the peptide complexed with the ACE2 protein (PEP(ACE2)), SPIKE, and the peptide complexed with the spike protein (PEP(SPIKE)). The RMSD results demonstrate that ACE2 and spike proteins exhibit minimal conformational changes, indicating their stability throughout the 100 ns of simulation. In contrast, despite its smaller size, the peptide displayed higher RMSD values in both simulations, indicating more remarkable conformational changes during the MD. This is attributed to the outstanding flexibility of the peptide, as it recursively rearranged itself to maintain the H-bonds and salt bridges. Finally, Figure 4C presents a 2D representation of the frames with the highest number of H-bonds and salt bridges during MD for the ACE2-PEP-S2 and SPIKE-PEP-S2 complexes, where the amino acids involved in chemical contacts are highlighted.

**Figure 5:**
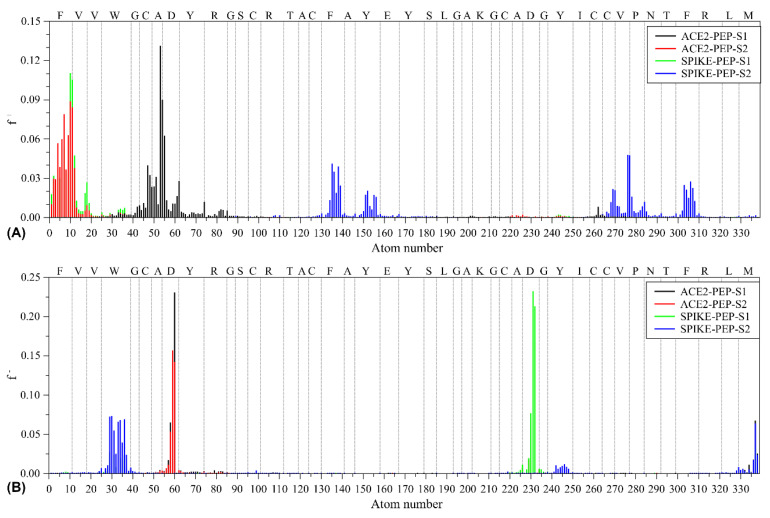
CAFI descriptors estimated for distinct conformation of peptide sequences. Peptide sites with reactivity towards (**A**) nucleophilic and (**B**) electrophilic agents.

Figure 5 illustrates the estimated condensed-to-atoms Fukui index (CAFI) values for the peptide sequence, where high values indicate regions with higher reactivity towards nucleophiles (*f*^+^) and electrophiles (*f*^−^). Aiming to investigate the influence of complex formation on the local reactivity of the resulting structures, the CAFIs were evaluated considering the geometries coming from molecular docking, without subsequent DFT-based geometry optimization, aiming to keep specific structural features of the adsorbed systems. It is worth noting the absence of negative CAFI values, suggesting the existence of plausible structures. Such analyses were conducted to investigate possible charge transfer processes that can take part during complex formation; in this sense, high CAFI values close to interaction centers suggest that the peptide adsorption can be with soft–soft interactions (via frontier molecular orbitals).

The dissimilarities observed between the structures can be associated with conformational variations. In general, it is evident that sites with high *f*^+^ values are close to donor residues (e.g., Phe_1_, Phe_18_, Val_37_, and Phe_41_), while sites with high *f*^−^ values are close to acceptor residues (e.g., Trp_4_, Asp_8_, Asp_31_, and Met_44_) identified in MD simulations. These findings further support the potential for effective interaction between the peptide and ACE2/spike structures.

All of these results revealed that stable complexes formed between the SS-I and ACE2/Spike protein. The stability of the complexes was explored through molecular dynamics simulations. Some studies demonstrated that the efficacy of ligands correlates with their residence time, which is also associated with their binding affinity [73]. Additionally, 100 ns (the simulation time used in this study) is commonly used to evaluate the complexes’ stability using a molecular dynamics approach [74]. In this way, our results indicated a high affinity between ligand and receptor and, consequently, the stability of the complexes (SS-I and ACE2/Spike) throughout the molecular dynamics simulations. However, despite all this evidence, it is not possible to state that these interactions occur in vitro, but it can be a good indication of potential ligands for experimental studies.

### 3.3. In Vitro Cytotoxic and Hemolytic Activity of Synthetic SS-I and SARS-CoV-2 Infection Assay

**Figure 6:**
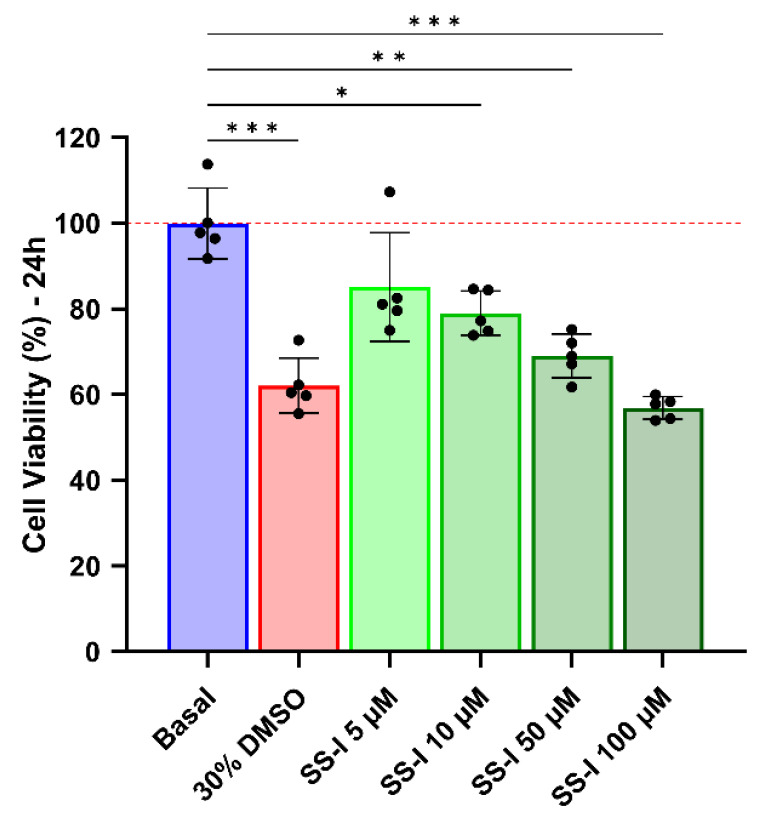
Viability measurement of C-20 microglial cells treated with synthetic SS-I at different concentrations using the MTT assay. The presented data represent the results of five independent experiments, with significant differences from the basal control group indicated and marked with asterisks (* *p* < 0.0332, ** *p* < 0.0021, *** *p* < 0.0002).

The initial step in determining the appropriate range of concentrations for an infection assay involves assessing the cytotoxicity of the SS-I synthetic peptide. Figure 6 demonstrates that a concentration of 5 μM, despite exhibiting an average viability of 85%, does not exhibit a significant difference compared to the basal condition.

The peptide at 10 μM showed a slight decrease in viability, with a significant difference from the basal control; however, 79% viability is not considered to indicate a cytotoxic effect [75]. Higher SS-I concentrations of 50 and 100 μM resulted in a decline in viability compared to that of cells treated with 30% dimethyl sulfoxide (DMSO).

**Figure 7:**
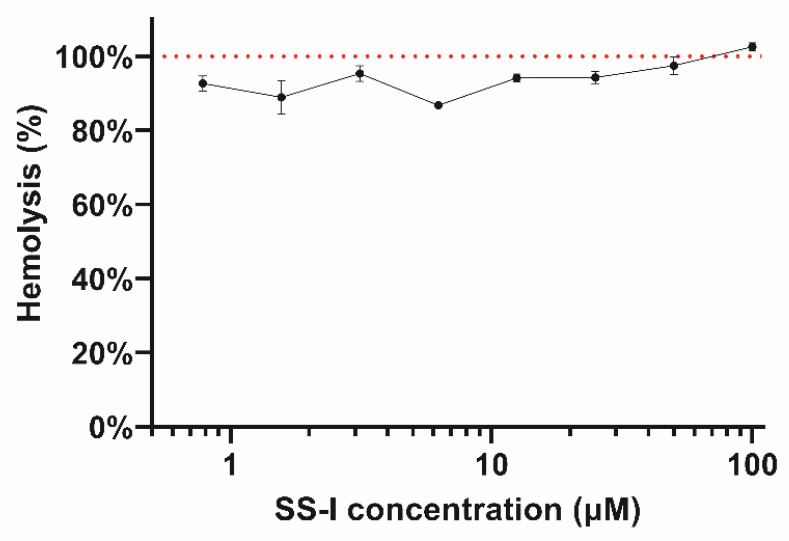
Hemolysis activity of SS-I synthetic peptide. Each concentration (0.78125 to 100 μM) was tested in triplicate. The red dotted line represents 100% hemolysis.

The hemolysis activity was assessed by measuring the release of hemoglobin at different peptide concentrations. Figure 7 illustrates that all concentrations between 100 and 0.78125 μM have a hemolytic effect. This peptide has a GRAVY index value of 0.339, which reveals it to have hydrophobic properties. The hydrophobicity in peptides has been linked to hemolytic effects due to the formation of pores in the cellular membrane [76,77,78]. The eukaryotic cell membrane, having a zwitterionic quality, is very susceptible to hydrophobic interactions [79]. Although the synthetic SS-I presents high hemolytic activity, it is not linked to high cytotoxicity in microglial cells, which can be explained by the high fragility of red blood cells [78,80]. The mechanisms through which the AMPs affect the cell membranes are still not fully understood and many have been proposed [81]. Nonetheless, the consensus is that the phospholipid composition of cell membranes determines how these peptides interact with cells [82,83,84]. A lower phosphocholine-to-sphingomyelin ratio, linked to low membrane fluidity, is associated with higher tolerance to membrane-disrupting activity [85]. The outer leaflet of the erythrocyte membrane comprises around 45% of phosphatidylcholine [86]. The observation of a high percentage may provide a potential explanation for the heightened hemolytic activity exhibited by SS-I in contrast to cytotoxicity.

**Figure 8:**
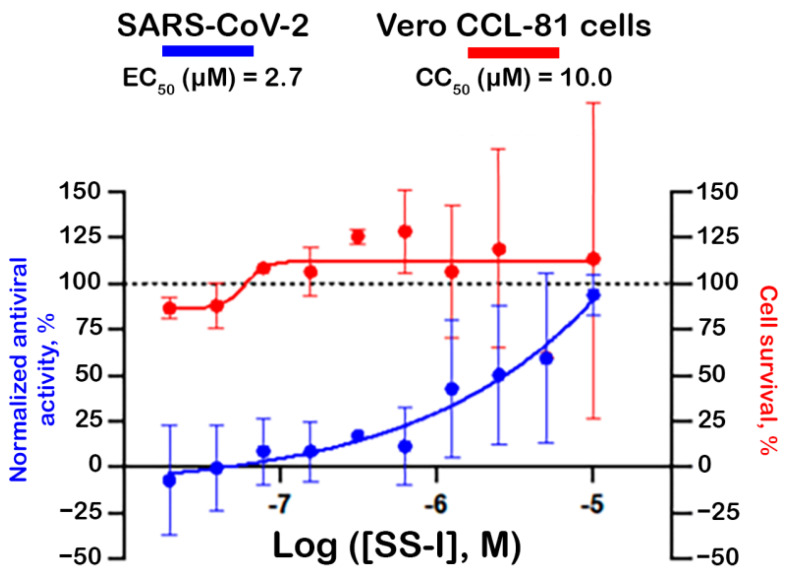
SARS-CoV-2 antiviral concentration–response and cell survival curves for synthetic SS-I. The fitted EC_50_ and CC_50_ values are the average of three independent experiments. The dotted line represents 100% antiviral activity and cell survival.

The inhibitory capacity of synthetic SS-I against the SARS-CoV-2 infection in Vero CCL-81 cells was assessed. As shown in Figure 8, the peptide demonstrated the ability to inhibit viral infection with an EC_50_ of 2.7 μM. The same assay showed a 50% cytotoxic concentration (CC_50_) of 10 μM in Vero CCL-81 cells. It is possible that, if higher concentrations of synthetic SS-I were tested, the CC_50_ could be higher as well. However, it should be noted that the higher tested concentrations have a greater associated error, which could have biased the CC_50_ determination. These results are consistent with the MTT assay, where a concentration of 10 μM was the highest at which the synthetic peptide did not exhibit a cytotoxic effect. Nevertheless, since the EC_50_ is lower than the calculated CC_50_, SS-I holds therapeutical relevance, and it is also viable to use concentrations that might have a more significant effect without jeopardizing cellular viability.

Considering that the linearized form of SS-I was demonstrated to have antiviral activity, we can infer that a fragment of SS-I can maintain the activity. The antimicrobial effect is associated with the peptide’s positive charge, while the cytotoxic and hemolytic activity, as already mentioned, is linked to hydrophobicity [76,77]. Besides the synthesis of smaller fragments being simpler and more cost-effective, it would enable the design and acquisition of a peptide with higher antimicrobial activity while reducing cytotoxic and hemolytic effects. Reynolds et al. demonstrated that defensin N-terminal fragments are potent AMPs [87], making this a promising next step for future works.

## 4. Conclusions

Alongside the development and implementation of vaccinations, it is crucial to invest in the study of novel front-line therapeutic strategies to fight viral diseases and reduce their associated morbidity and mortality. The research and characterization of new peptides, either as standalone treatments or as adjuncts to existing antiviral and antibacterial drugs, hold significant strategic importance in equipping us with tools for the prompt therapeutic development and management of viral and bacterial infections.

The field of antimicrobial resistance is dynamic and rapidly evolving, posing ongoing challenges for clinicians in the treatment of resistant infections.

In this study, we identified a novel defensin, SS-I, identified from the tail-tip of the salamander *S. salamandra*. A BLAST search revealed that SS-I exhibited the highest similarity to CFBD-1, a salamander defensin previously identified in *C. fudingensis*.

All simulations and interaction studies were implemented using the predicted tridimensional peptide structure. The performed in vitro assays, on the other hand, most likely used the peptide in its linear form. Even though the synthesized peptide exhibited antiviral activity, we cannot assert that the predicted interactions between the peptide and ACE2 and spike proteins are responsible for the observed infection inhibition. For that, additional studies, such as nuclear magnetic resonance (NMR) and X-ray crystallography, are needed to fully characterize the peptide structure and its interaction with ACE2 and spike proteins. Moreover, we can conduct regio-specific disulfide bond formation in order to perform structure–activity studies. Such studies can provide a deeper understanding of the antiviral activity of the peptide and its potential use in the development of new therapeutic agents against viral infections.

The synthesized peptide proved to be highly hemolytic at every tested concentration but not cytotoxic in the antiviral concentration range. As prospects, the research of SS-I-derived fragments could overcome the hemolytic effects and further improve their antimicrobial activity while being a more accessible synthesis.

There is currently extensive nonclinical and clinical research underway to evaluate the potential of AMPs as next-generation antibiotics. Advancements in areas such as smart formulation strategies, advanced chemical synthesis protocols, and a deeper understanding of their mechanism of action are expected to accelerate the translation of AMP-based research findings into viable pharmaceutical product candidates. This peptide could be used as a tool to study the interaction between the virus and host cells, which could contribute to the understanding of the pathogenesis of COVID-19 and the development of new therapeutic strategies. Further investment in research and development is necessary to fully assess the potential of these novel and effective tools.

In conclusion, besides the development of vaccinations, it is essential to focus on exploring new therapeutic strategies and antimicrobial peptides to combat viral and bacterial infections effectively. These efforts will contribute to the advancement of therapeutic options and help address the challenges posed by antimicrobial resistance.

## Figures and Tables

**Table 1:** Residues in ACE2-PEP-S2 and SPIKE-PEP-S2 complexes with H-bonds and salt bridges of >10% occupancy throughout MD simulation.

ACE2-PEP-S2			SPIKE-PEP-S2		
H-Bonds					
Donor	Acceptor	Occupancy	Donor	Acceptor	Occupancy
Ala_16_-PEP	Asp_30_-ACE2	93.72%	Arg_42_-PEP	Glu_484_-SPIKE	97.74%
Thr_15_-PEP	Asp_30_-ACE2	93.28%	Thr_500_-SPIKE	Asp_31_-PEP	30.33%
Lys_26_-ACE2	Asp_31_-PEP	65.47%	Phe_1_-PEP	Asn_501_-SPIKE	22.97%
Gly_5_-PEP	Ala_387_-ACE2	64.17%	Arg_42_-PEP	Gly_482_-SPIKE	20.09%
Arg_42_-PEP	Glu_37_-ACE2	62.71%	Phe_1_-PEP	Gln_498_-SPIKE	19.83%
Arg_14_-PEP	Asp_30_-ACE2	62.67%	Asn_450_-SPIKE	Met_44_-PEP	19.13%
Trp_4_-PEP	Gln_388_-ACE2	36.05%	Asn_39_-PEP	Glu_484_-SPIKE	17.19%
Lys_353_-ACE2	Leu_43_-PEP	27.77%	Gln_498_-SPIKE	Asp_31_-PEP	14.91%
Tyr_33_-PEP	Asp_30_-ACE2	15.81%	Asn_501_-SPIKE	Phe_1_-PEP	14.73%
Trp_4_-PEP	Ala_387_-ACE2	15.35%	Tyr_33_-PEP	Gln_498_-SPIKE	12.20%
			Thr_15_-PEP	Gly_446_-SPIKE	11.32%
			Asn_39_-PEP	Glu_484_-SPIKE	10.58%
**Salt Bridges**					
**Residues**		**Occupancy**	**Residues**		**Occupancy**
Asp_31_-PEP/Lys_26_-ACE2		82.18%	Glu_484_-SPIKE/Arg_42_-PEP		93.90%
Asp_30_-ACE2/Arg_14_-PEP		68.21%			
Glu_37_-ACE2/Arg_42_-PEP		61.13%			

## Data Availability

The data presented in this study are available within the article and Supplementary Material.

## Supplementary Materials

The following supporting information can be downloaded at https://www.mdpi.com/article/10.3390/pharmaceutics16020190/s1, Figure S1: ACE2-PEP-S1, ACE2-PEP-S2, SPIKE-PEP-S1, and SPIKE-PEP-S2 molecular complexes obtained through docking analysis and used for MD simulations. Figure S2: MS/MS Spectrum of the triply charged ion [M + 3H]^3+^ of SS-I.
