# Supplementary material for: Antiviral Action against SARS-CoV-2 of a Synthetic Peptide Based on a Novel Defensin Present in the Transcriptome of the Fire Salamander (Salamandra salamandra)
Source: Pharmaceutics. 2024 Jan 29;16(2):190. doi: 10.3390/pharmaceutics16020190 (PMC10892092; doi:10.3390/pharmaceutics16020190)
Supplement: Supplementary file 1 [file pharmaceutics-16-00190-s001.zip › pharmaceutics-2798638-supplementary.pdf]

Supporting Information

Antiviral Action against SARS-CoV-2 of a Synthetic Peptide Based on a Novel Defensin Present in the Transcriptome of the Fire Salamander (*Salamandra salamandra*)

Ana Luisa A. N. Barros [1,2], Vladimir C. Silva [3], Atvaldo F. Ribeiro-Junior [1], Miguel G. Cardoso [1,4], Samuel R. Costa [5], Carolina B. Moraes [6], Cecília G. Barbosa [7], Alex P. Coleone [8], Rafael P. Simões [9], Wanessa F. Cabral [1], Raul M. Falcão [10], Andreanne G. Vasconcelos [1,11], Jefferson A. Rocha [12], Daniel D. R. Arcanjo [13], Augusto Batagin-Neto [8,14], Tatiana Karla S. Borges [1], João Gonçalves [4], Guilherme D. Brand [5], Lucio H. G. Freitas-Junior [7], Peter Eaton [15,16], Mariela Marani [17], Massuo J. Kato [18], Alexandra Plácido [15] and José Roberto S. A. Leite [1,*]

[1] Núcleo de Pesquisa em Morfologia e Imunologia Aplicada, NuPMIA, Faculdade de Medicina, Universidade de Brasília, UnB, Brasília 70910-900, DF, Brazil
[2] Programa de Pós-graduação em Medicina Tropical, PGMT, Faculdade de Medicina, Universidade de Brasília, UnB, Brasília 70910-900, DF, Brazil
[3] Laboratório de Vigilância Genômica e Biologia Molecular-Fundação Oswaldo Cruz Piauí, Teresina, PI 64001-350, Brazil
[4] imed.ULisboa-Research Institute for Medicines, Faculty of Pharmacy, University of Lisbon, 1649-003 Lisbon, Portugal
[5] Instituto de Química, IQ, Universidade de Brasília, UnB, Brasília 70910-900, DF, Brazil
[6] Department of Pharmaceutical Sciences, Federal University of São Paulo, Diadema 09913-030, SP, Brazil
[7] Department of Microbiology, Institute of Biomedical Sciences, University of Sao Paulo, São Paulo 05508-000, SP, Brazil
[8] Programa de Pós-graduação em Ciência e Tecnologia de Materiais (POSMAT), School of Sciences, São Paulo State University (UNESP), Bauru 17033-360, SP, Brazil
[9] School of Agriculture, Department of Bioprocess and Biotechnology, São Paulo State University (UNESP), Botucatu 18618-689, SP, Brazil
[10] Bioinformatics Postgraduate Program, Metrópole Digital Institute, Federal University of Rio Grande do Norte, Natal 59078-900, RN, Brazil
[11] People&Science Pesquisa Desenvolvimento e Inovação LTDA, Centro de Desenvolvimento Tecnológico (CDT), Universidade de Brasília, UnB, Brasília 70910-900, DF, Brazil
[12] Campus São Bernardo, Universidade Federal do Maranhão, UFMA, São Bernardo 65550-000, MA, Brazil
[13] Department of Biophysics and Physiology, Federal University of Piauí, Teresina 64049-550, PI, Brazil
[14] Institute of Sciences and Engineering, São Paulo State University (UNESP), Itapeva 18409-010, SP, Brazil
[15] Laboratório Associado para a Química Verde/Rede de Química e Tecnologia (LAQV/REQUIMTE), Departamento de Química e Bioquímica, Faculdade de Ciências, Universidade do Porto, 4169-007 Porto, Portugal
[16] School of Chemistry, The Bridge, University of Lincoln, Lincoln LN6 7EL, UK
[17] IPEEC-CONICET, Consejo Nacional de Investigaciones Científicas y Técnicas, Puerto Madryn 9120, Argentina
[18] Instituto de Química (IQ), Universidade de São Paulo (USP), São Paulo 05508-900, SP, Brazil
* Correspondence: jrsaleite@gmail.com

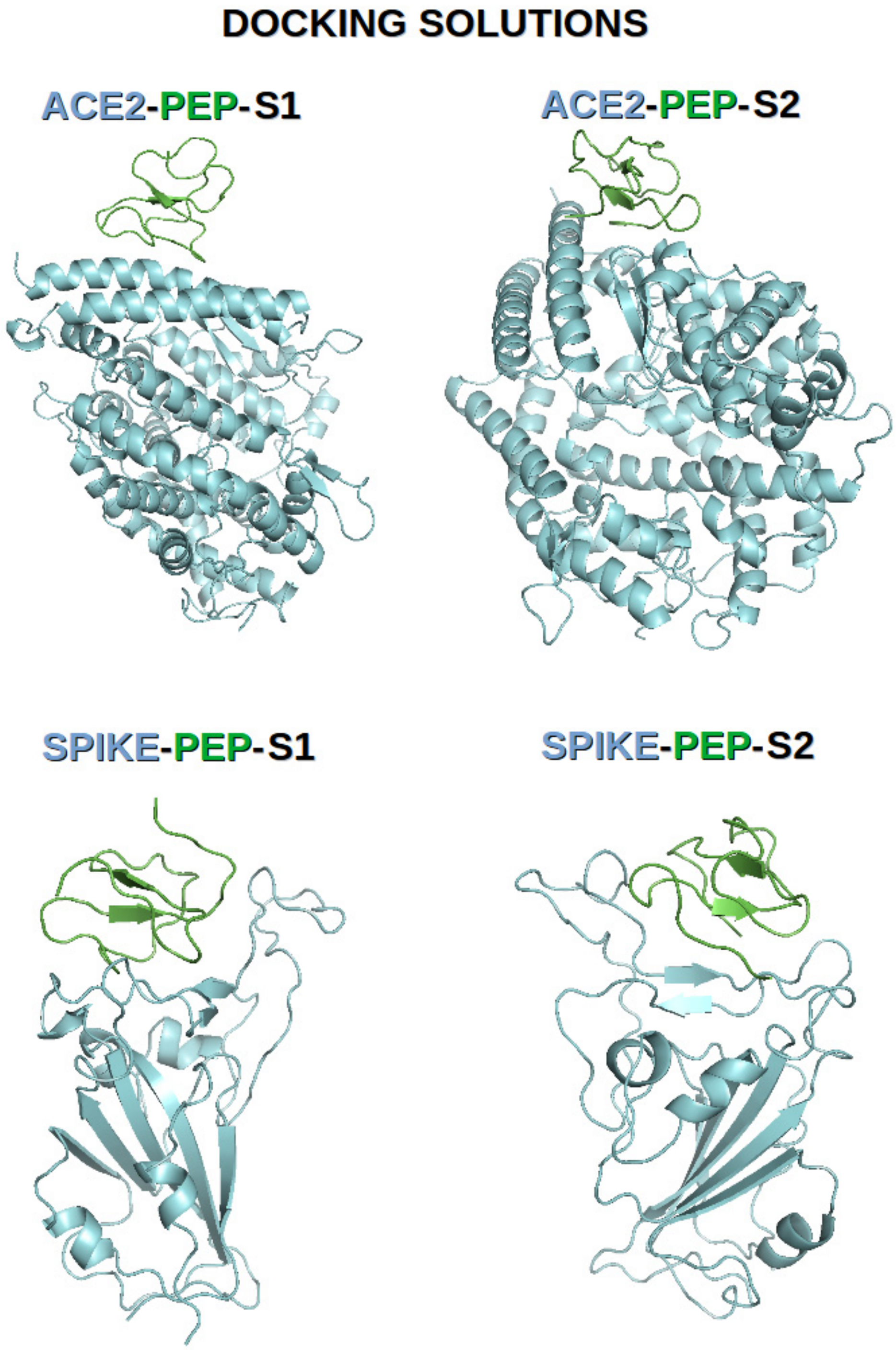

Figure S1. ACE2-PEP-S1, ACE2-PEP-S2, SPIKE-PEP-S1, and SPIKE-PEP-S2 molecular complexes obtained by docking analysis and used for MD simulations.

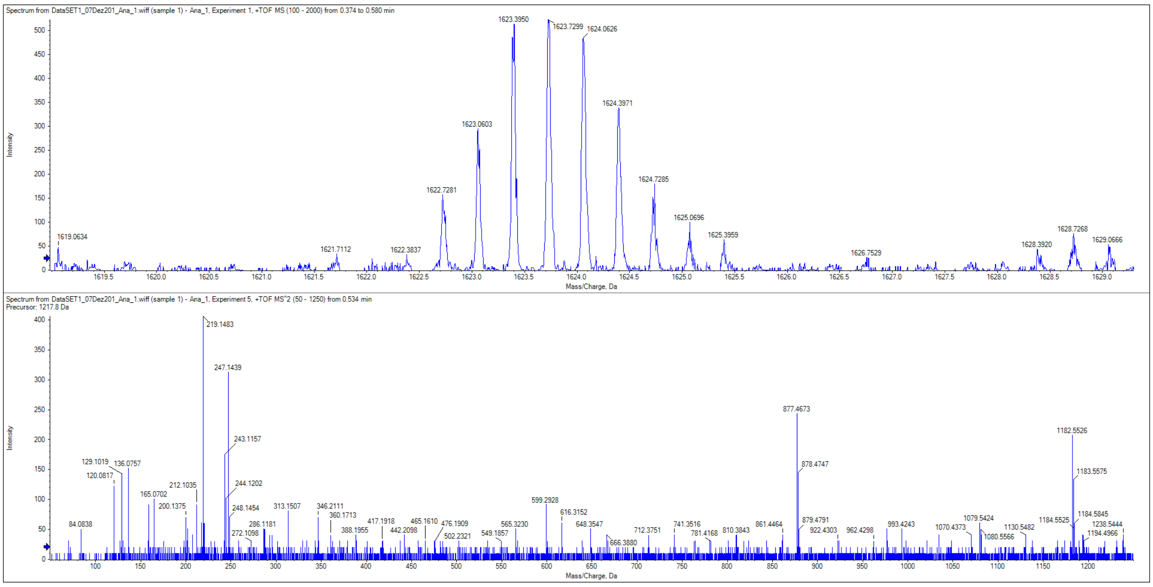

Figure S2. MS/MS Spectrum of the triply charged ion $[M+3H]^{3+}$ of SS-I.
